# Humanoid Identification of Fabric Material Properties by Vibration Spectrum Analysis

**DOI:** 10.3390/s18061820

**Published:** 2018-06-05

**Authors:** Shuyang Ding, Yunlu Pan, Xuezeng Zhao

**Affiliations:** Key Laboratory of Micro-Systems and Micro-Structures Manufacturing, Ministry of Education and School of Mechatronics Engineering, Harbin Institute of Technology, Harbin 150001, China; 14b308001@hit.edu.cn (S.D.); zhaoxz@hit.edu.cn (X.Z.)

**Keywords:** textile discrimination, fabric sensation, tactile perception, vibration, roughness, hardness

## Abstract

In daily contexts, fabrics embodied in garments are in contact with human body all the time. Since fabric material properties—such as softness or fineness—can be easily sensed by human fingertips, fabric materials can be roughly identified by fingertip sliding. Identification by simply touching and sliding is convenient and fast, although the room for error is always very large. In this study, a highly discernible fabric humanoid identification method with a fingertip structure inspired tactile sensor is designed to investigate the fabric material properties by characterizing the power spectrum integral of vibration signal basing on fast Fourier transform integral *S*(*FFT*), which is generated from a steel ball probe rubbing against a fabric surface at an increasing sliding velocity and normal load, respectively. *kv* and *kw* are defined as the slope values to identify the fabric surface roughness and hardness. A sample of 21 pieces of fabric categorized by yarn weight, weave pattern, and material were tested by this method. It was proved that the proposed humanoid sensing method has more efficient compared with fingertip sliding while it is also much more accurate for fabric material identification. Our study would be discussed in light of textile design and has a great number of potential applications in humanoid tactile perception technology.

## 1. Introduction

Touch is an interactive sense in feeling objects, which is unique from the senses of vision and hearing. Sights and sounds can be observed without physical interaction, while the tactual properties of an object can only be sensed by physical contact. Though vision has a high spatial resolution and preserves fine details, tactile sensation on exploring and distinguishing material properties is much better than vision [1]. People decide to purchase textile products based on their tactile perception knowledge during the interaction with the textile fabric surfaces by fingertip sliding [2], because textures like smoothness, glossiness, and naturalness as stimuli can be sensed by a great of mechanoreceptors in the human skin. These sensory receptors respond to mechanical and thermal stimuli, which include the Pacinian corpuscle, a primary receptor that senses vibration with a range of 60 to 700 Hz [3,4,5]; the Meissner corpuscle, which is sensitive to the contour structure of surfaces; the Merkel corpuscle, which can sense the constant contact irritant on to skin; the Ruffini ending which is located in the deep epidermis and responds to tensile changes caused by the skin friction between the contact surfaces of skin and fabric [6,7,8,9,10]. Stimuli of temperature or tickling can also be observed by some other receptors in our skin [11]. In human tactile perception process, skin acts both a force transmitter and a sensor in interacting with the surfaces we touch [12,13]. Intensive research demonstrated that the tactile perception of various physical stimuli is generated by fingertip sliding along the surface, which permits identification and/or discrimination of textural properties. For instance, when the skin deformation and friction induced vibration stimulate the sensory receptors, the surface texture information is transmitted into a potential current and delivered to human brain by nerve fibers as we presented in the previous studies [14].

The literature shows that stimuli of textile fibers in subjective tactile perception studies basically include: hard/soft, rough/smooth, fine/coarse, sticky/slippery, cold/warm, and so on [15,16,17,18,19,20,21]. Numbers of factors proved to be influential: fiber type (e.g., natural or artificial, etc.), yarn weight, production method (e.g., fabric weave pattern, etc.), type of dyeing, and finishing process (i.e., heat treatment, brushing, coloring, softening, etc.) [22,23]. Krista et al. investigated the tactile perception of naturalness in 44 pieces of textiles made of wool, cotton, acrylic, and polypropylene, 32 participants took part in the textile estimation and present a subjective identification in four different methods [24]. Bergmann and Kappers studied on 125 kinds of surfaces and found that four dimensions are needed at least to describe objects surfaces [25]. Bolanowsku et al. proposed that the tactile sense for most objects can be identified in their hardness, roughness, and an uncertain dimension [26]. However, human subjective sensing experiments on fabrics are performed in terms of time and cost. Thus, an objective method is necessary for conducting an efficient and accurate textile fabric identification. For fabric surface texture perception, computerized image processing techniques—such as confocal microscope, scanning electoral microscope, etc.—are widely applied to characterize and evaluate fabric surfaces quantitatively both in the textile industry and academic research [27,28,29]. The visual method is usually used to obtain fine details of the fiber surface, while some textile fabric material properties can only be obtained by touch sensing, such as smoothness or hardness. Other objective methods are usually performed by collecting digital signals generated from the interaction surface between a tactile sensor and the textile fabric surface, then the signals are converted and analyzed by an intelligent tactile perception system assessment to identify the fabric properties [30]. Tactile sensors can accurately perform more functionally than human fingertips, especially in evaluating fabric surface textures and material properties, which leads to a great benefit to textile product quality as well as reducing the cost of production and inspection. Hu et al. developed a finger-shape tactile sensor to evaluate fabric surface properties [31]. Koc and Aksu investigated the constructional differences in fabrics with a polymeric fingertip [32]. Kikuuwe et al. investigated the fine surface properties of textile fabrics with a finger mounted tactile sensor [33]. Chen at al. studied the intelligent identification of fabric texture properties using a biotech tactile sensor [34]. Hollins et al. found evidence for a duplex theory of tactile texture perception [35]. Moreover, the frictional stimuli between the skin and the contact surfaces has been caught great attention in quantifying the tactile perception of textile fabric textures. However, the friction coefficient between human skin and textile fabric is usually affected by various factors such as humidity, sliding velocity, and the properties of the skin itself [36,37,38,39,40]. Pailler-Mattei et al. [41] have shown that the lipidic film on the skin surface can be related to the skin adhesion and the kinetics of sorption/desorption of distilled water by the skin will affect the skin friction coefficient. Bhushan et al. demonstrate the mechanism of influence of sliding velocity on shear strain rates, which results in a lower real area of contact and a lower coefficient of friction in a dry contact [42]. According to the literature, it is remarkable that there has not been any thorough study into the identification of fabric material properties in vibration spectrum analysis compared with human sensing.

**Figure 1:**
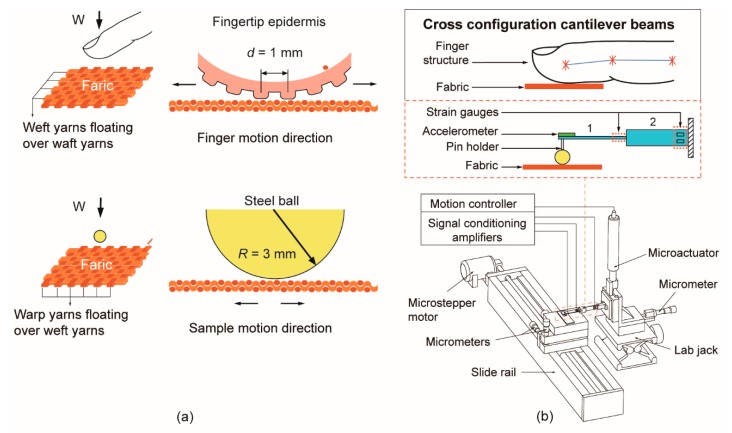
(**a**) Schematic illustrating the processes of human tactile perception by an index fingertip and a steel ball probe, when they are sliding on the surface of 1/1 plain weave fabrics consisting of weft and warp yarns in each direction. (**b**) A finger biological structure-inspired tactile sensor with cross configuration cantilever beams was designed and mounted on a reciprocating motion tribometer to access the tactile perception [12,14].

In this paper, we first investigated human fingertip tactile perception on fabric properties. A sample of 21 different commercial textile fabric substrates were varied by yarn weight, weave pattern, and fabric material (pure and mixed materials). Then, 45 participants were asked to explore the identification of these textile fabrics on rough/smooth, hard/soft, and pleasant/unpleasant feeling during an index finger sliding in weft yarn direction as shown in Figure 1a. A cross-configuration cantilever beam tactile sensor was designed with a steel ball mounted at the free edge to investigate textile fabric identification as the steel ball rubbed against the fabric surface with an increasing sliding velocity and normal load. The tactile sensor was inspired by the biological bone structure of the human index finger and the measurement setups were designed to mimic the human touch behaviors with different velocities and preloads as shown in Figure 1b. Then friction force and acceleration signals are collected and converted into the friction coefficient and power spectrum for the tactile identification on fabrics.

A simple and effective fabric humanoid identification method basing on the previous research [14] was proposed by characterizing the power spectrum integral *S*(*FFT*^2^) of the vibration signal in the frequency domain. Fabric textures were identified by the slope values of *S*(*FFT*^2^) and *S*(*FFT*) as a function of sliding velocity and normal load, which were defined as *kv* and *kw* respectively for surface roughness and hardness. This humanoid identification method provides a wider discrimination range and a higher discernible resolution than human fingertip sensations on fabric material properties (over 90% of the fabric substrates were significantly discriminated). The aim of this research is to achieve a biomimic method on fabric material property identification by vibration spectrum analysis compared to human fingertip sensation. It would be a potential look into humanoid robot tactile sensing abilities.

## 2. Experimental and Method

### 2.1. Fabrics Samples

Twenty-one different pieces of commercial textile fabrics manufactured by WHALEYS (BRADFORD) LTD (West Yorkshire, UK) were used in this study, which include 6 pieces of cotton canvas fabrics with yarn weight ranging from 3.5 to 15 oz/yd^2^; 4 pieces of cotton fabrics with different weave patterns of canvas, gauze, drill and satin; 11 pieces of plain woven fabrics made by different materials—which include 5 natural materials of cotton, silk, wool, nylon, and acetate; 6 mixed materials of cotton and silk, cotton and elastane, cotton and polyethylene, silk and wool, silk and elastane, and silk and lycra. All these textile fabrics were trimly cut into 80 × 25 mm rectangle shape samples and attached on microscope slides (Fisher brand^®^, plain beveled edge, 80 × 25 × 1 mm) with double sided tape (3M 9080HL). The microscope photographs of the 21 pieces of fabric substrates are conducted by a digital microscope (Leica DVM 6, Buffalo Grove, IL, USA).

### 2.2. Participants

Thirty-five participants (20 males, 15 females; 33 right-handed, 2 left-handed; mean age 25.6, range 17–35) took part in this study. All participants had normal vision, normal touch and no experts concerning textiles. The participants sat behind a table in front of a 80 cm × 80 cm × 80 cm photographic LED-light tent (Hakutatz, TB800C, Shanghai, China) as illustrated in the supporting information Figure S1. A white plastic sheet was laid in the tent to exclude the possible influence of background vison. During the testing of samples with their index finger, each participant had noise canceling headphones on to eliminate the influence of acoustic cues. Hands were washed with glycerin soap and dried with paper towels before the task and whenever they needed in the exploration process. All the tactile explorations were carried out in the same ambient conditions (22 °C, RH 35–40%).

### 2.3. Experimental Sprocedure and Method

First, in human subjective fingertip tactile perception tests, all fabric substrates were firmly mounted on glass slides, then divided into three groups and attached in a row with double sided tapes on three plastic stages with dimension of 20 cm × 10 cm × 1 cm. Each stage was placed in the center of a daylight tent and illuminated in a constant lighting condition of 60 W with 120 LEDs inset of the top. Three kinds of fabric texture tactile perceptions were investigated in this test—which included yarn weight, weave pattern, and material properties—and fabric substrates were selected and placed into three groups by textures. For conducting an effective comparison and reducing the memory work in the human fingertip tactile perception, each group possessed, at most, six pieces of textile fabric substrates. Visual explorations were allowed in the test with a distance of approximately 40 cm and tactile explorations were conducted with only the index finger of the subjects’ dominant hand. Then, the participants were asked to explore the tactile perception of the fabric textures in all groups by conducting a movement of reciprocating sliding with freely pressure and scanning velocity to discriminate the fabric surface textures within 5 min, then ranking the samples from most to least according to their subjective sensation of rough/smooth, hard/soft, and pleasant/unpleasant. The participants can change the order of the stimuli at any time during the tasks. When they finally reached their ranked order in one group, the samples were scored by ranking from 0 to 6 corresponding to the position in the row from left to right. For each group, the participant had to complete three tactile explorations for repetitive purposes. From the beginning of the task, we intentionally randomized the order of the fabric samples in every group to exclude memory effects on participants during the three tactile explorations. Thus, we averaged the tactile perception scores of these three groups of textile fabrics and, finally, the results were compared with the fabric physical properties of roughness and hardness. 

Next, for the objective perception by tactile sensor, 21 textile fabrics samples in groups of three, according to their texture properties, were tested through scanning against a steel ball (diameter = 6 mm, *Ra* = 0.05 µm) which was mounted on a pin-holder and screwed at the edge of a fingertip inspired cross-configuration cantilever beam. The dimensions of the probe were determined by the nominal contact area between the fabric substrate and steel ball surface. Then the vibration signal was collected by a small, low profile (4 mm × 4 mm × 1.45 mm, mass 1.27 g) three-axis accelerometer (Analog Devices, ADXL335, Norwood, MA, USA) which has a full-scale range of ±3 g, the bandwidth has a range of 0.5 Hz to 1.6 kHz in the *x* and *y* directions and 0.5–550 Hz in the *z* direction; and sensitivity at *x*, *y*, and *z* of 300 mV/g ± 0.001 mV/g. Two strain gage force sensors with a gauge factor of 115 were installed on each cantilever respectively, as shown in the schematic of the experimental set-up in Figure 1b. The natural frequency of the beam is around 5 kHz above the measured frequency of 0 to 1000 Hz. Acceleration, friction force, and normal load signals are collected at a sampling frequency of 4 kHz, considering a frequency measurement range of 0–1 kHz is sufficient to measure all the dominant frequencies of vibration in the present experimental conditions. These parameters were digitized with a resolution of 12 bits in the range of 3.5 V through LabVIEW 2016 software (National Instrument™, Austin, TX, USA) and onboard electronics. Then, spectral analyses of the signals were conducted by fast Fourier transform and coherence function in MATLAB version R2016a.

In this study, the textile fabric substrate was attached on the sliding stage as shown in Figure 1b, 21 fabric substrates were grouped by yarn weight, weave pattern, and material, then the friction coefficient and spectral analysis are performed with sliding velocities increasing from 5 mm/s to 40 mm/s and normal loads ranging from 20 mN to 100 mN, respectively. The vibration signal spectrum analysis of all the fabrics was systematically compared with the fabrics physical properties of roughness and hardness. The roughness (*Ra*) of the fabrics were measured and conducted by Leica DVM 6 and Leica MAP software. The hardness of the fabrics was measured by Shore Durometer (Elecall, LXD-C) which is applicable to colloid and fabric materials. The average roughness and hardness were obtained by calculating the mean of five times measurements. All the physical properties of fabrics were listed in Table 1 and Table 2.

In order to figure out a simple and reliable approach to identify textile fabrics by tactile perception. We proposed a novel method to conduct the identification of fabric texture in hardness and roughness by characterizing *S*(*FFT*) the frequency spectral integral and *S*(*FFT*^2^) the power energy of the vibration acceleration signal in frequency domain during the interaction between a steel ball probe and fabric surface, which are defined as the following relationships:

(1) $$S\left(FFT\right)\;=\;\frac{b-a}{2N}\overset{N}{\underset{i\;=\;1}{\sum }}\left[FFT\left(f_{i}\right)+FFT\left(f_{i+1}\right)\right]$$

(2) $$S\left(FFT^{2}\right)\;=\;\frac{b-a}{2N}\overset{N}{\underset{j\;=\;1}{\sum }}\left[{\left(FFT\left(f_{j}\right)\right)}^{2}+{\left(FFT\left(f_{j+1}\right)\right)}^{2}\right]$$

where *S*(*FFT*) is the integral of *FFT*(*f*) and *S*(*FFT*^2^) is the integral of (*FFT*(*f*)^2^, *FFT*(*f*) is the fast Fourier transform (*FFT*) of the variable *x*(*t*) in time domain, *i* and *j* are ranged from 0 to *N*, and *N* is the number of sampling points, the spacing between each point equals to the scalar value $\frac{b-a}{N}$. Then *kw* and *kv* were defined as the characterizations of *S*(*FFT*) and *S*(*FFT*^2^) as a function of normal load and sliding velocity respectively, which were conducted in the following fabric surface texture tactile perception tests of different yarn weights, weave patterns, and materials.

## 3. Results and Discussions

### 3.1. Fingertip Sensation Test of Fabric Textures

**Figure 2:**
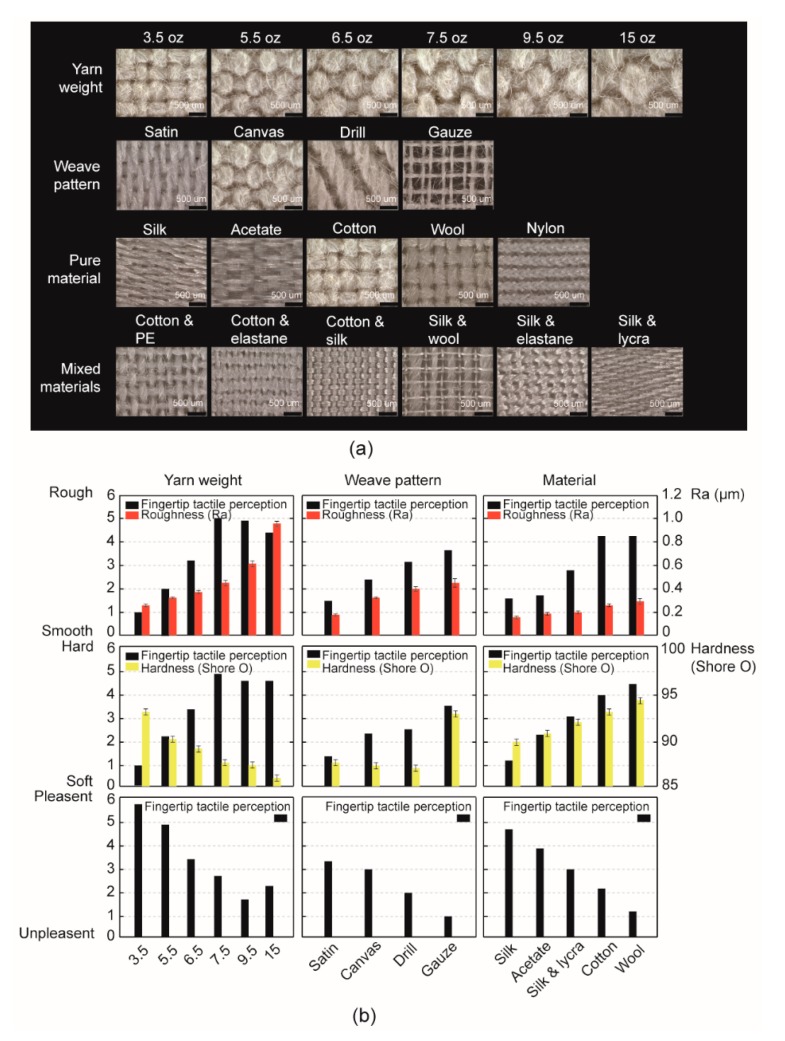
(**a**) A schematic overview of the stimuli from three groups of commercial textile fabrics used in the experiments. In the top, cotton fabrics with six different yarn weights from 3.5 to 15 oz/yd^2^ are shown. Then four different weave structures followed with five pure-material and six mixed-material fabrics are shown in the following panel. (**b**) Results for the yarn weight, weave pattern, and material tactile perception on rough/smooth, hard/soft, and pleasant/unpleasant. The black chart represents the mean estimation of human fingertip tactile perception. Other color charts illustrate the material properties as shown in the Table 1 and Table 2.

In this section, 15 pieces of fabric samples were selected basing on the fabric textures, which included 6 pieces of cotton canvas substrates with yarn weight from 3.5 to 15 oz/yd^2^; 4 pieces of cotton fabrics with different weave patterns of stain, canvas, drill and gauze; and 5 pieces of pure or mixed materials of cotton, silk, wool, acetate and silk mixed with lycra. Then, the repeated fingertip tactile perception of rough/smooth, hard/soft, and pleasant/unpleasant estimation was presented in Figure 2b. The black color bars represented the fingertip tactile perception of rough/smooth, hard/soft, and pleasant/unpleasant during the fingertip sliding on the fabric surface, which were scored from 0 to 6. Then, comparing these findings with the human subjective sensation results, the red and yellow color bars revealed the fabric physical properties of roughness and hardness which were measured by microscope and durometer, respectively.

In the yarn weight column on the left, we found significant interactions between the tactile perception and fabric material properties. As the roughness parameter in the red bars increased, the fine yarn fabric weights of 3.5 to 7.5 oz/yd^2^ presented an increasing linear dependence on the roughness properties, while fabrics with thick yarn ranging from 7.5 to 15 oz/yd^2^ demonstrated a decreasing trend on fingertip tactile perception, which means the participants were not able to discriminate these three thick-yarn fabrics simply relying on surface roughness only during the fingertip scanning on fabric surfaces. Then, as the yarn weight increased from 3.5 to 15 oz/yd^2^, the hardness bars in the yellow decreased linearly, however, the fingertip tactile perception presented an opposite regularity according to the fabric hardness sensation, which proved that the sensitivity of human fingertip on fabric hardness discrimination was far from the fulfillments to fabric yarn weight identification. The pleasant/unpleasant estimation of different yarn weights in the bottom revealed a comprehensive sensation which was mainly determined by roughness than that was by hardness. In the second column of the weave pattern test, a linear coherence between the fingertip tactile perception and roughness properties was shown, while the hardness properties measured on the different weave patterns of satin, canvas, and drill in yellow bars appeared to be not consistent with the human tactile perception results. However, in the pleasant/unpleasant comparison, we found smoother and softer fabrics basically provided a more pleasant sensation basing on different weave patterns as shown in the black color bars. Finally, in the third column of fabric material sensation, it showed two increasing trends on both rough/smooth and hard/soft tactile perceptions. Furthermore, we found materials such as silk, acetate, and silk mixed with lycra—which had similar surface roughness properties but different material hardness—revealed an obvious discrimination in pleasant sensation. Above all, the explorations on the fabric tactile perception indicated that the human subjective sensation on fabric texture basically can identify the different textures, while it was less sensitive in identifying some fine-yarn fabrics. 

### 3.2. Signals on Fabric Textures Obtained by the Tactile Sensor

Friction force, normal load, and acceleration signals were generated during a steel ball sliding on 21 different fabric substrates in the weft yarn direction. The spectral analysis with *FFT* and coherence function was applied in the fabric texture discrimination study. Friction coefficient and spectral analysis were compared as function of sliding velocity and normal load respectively for conducting a new method on fabric tactile perception and texture identification. 

**Figure 3:**
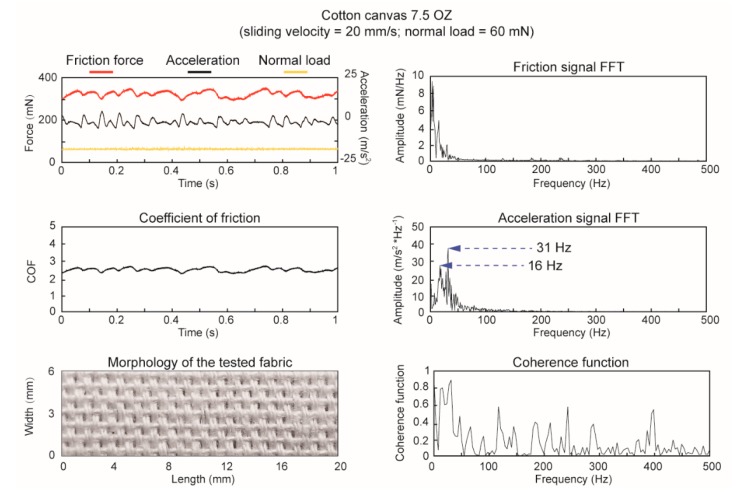
Friction force, acceleration, and normal load signals during a steel ball probe sliding on 7.5 (oz/yd^2^) cotton canvas fabric at 20 mm/s scanning velocity under 60 mN preload are acquired. The spectra analysis of fiction force and acceleration by fast Fourier transform (*FFT*) were present and the coherence function analysis illustrated the relationship between the two signals.

Figure 3 demonstrated three synchronously measured signals in time domain (in a one-second scope), which were collected from a cotton canvas fabric rubbing against a steel ball with a sliding velocity of 20 mm/s and a preload of 60 mN (the sliding velocities and normal loads were applied equally to that in human fingertip sensation tests). Then the friction coefficient was conducted in real-time and the morphology of the tested plain woven sample were shown in the bottom, which provided a periodical stimuli from the fabric structure of 16 warp yarns floating over the weft yarns. The spectral analysis and coherence function of friction force and acceleration signals were shown on the right, which indicated two high correlations of friction force and acceleration signals with the peak values of 16 Hz and 31 Hz, respectively. These frequency spectral peaks were able to reflect some information of the fabrics surface, such as the weave pattern or waviness, while it was still hard to describe the fabric textures of roughness or hardness.

Based on hundreds of fabric experimental analyses, we proposed a new method for identifying the fabric textures by characterizing the changing rate of power spectrum integral as a function of sliding velocities and normal load respectively, as we presented in the method section. In this study, fabrics with different yarn weights, weave patterns, and materials were able to be discriminated simply by values of *kv* and *kw*, which represented fabric surface roughness and hardness. The experimental results of the fabric material properties identification test were shown in following figures and the *kv* and *kw* values were listed in Table 1 and Table 2.

### 3.3. Yarn Weight Tactile Perception

**Figure 4:**
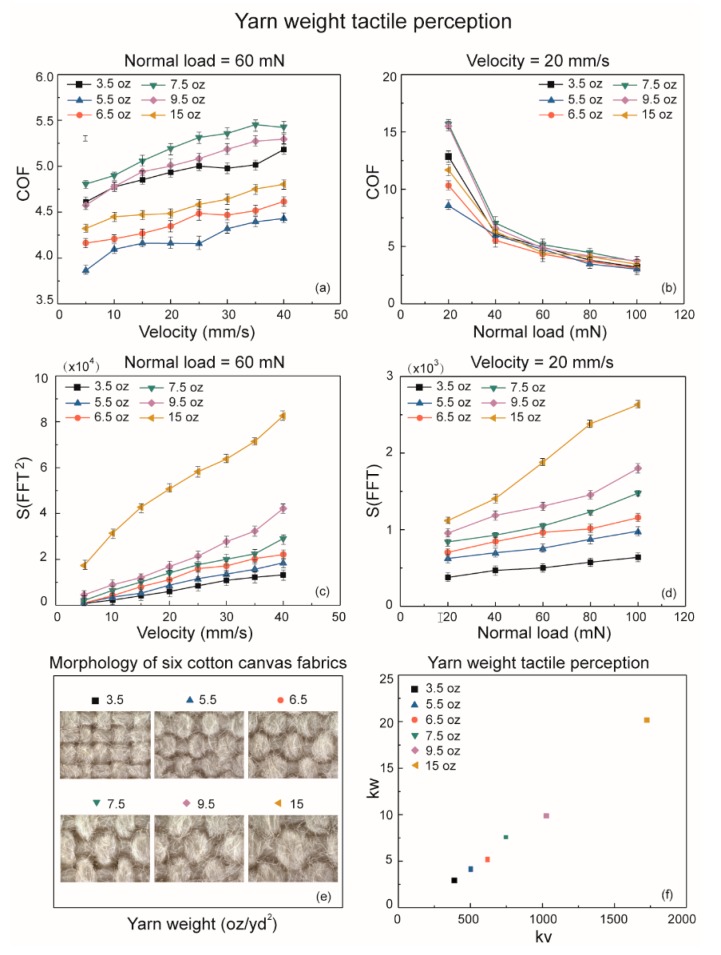
The frictional properties and tactile perception yarn weigh (oz/yd^2^) as a function of velocity and normal load on cotton canvas textile fabrics. (**a**) Effect of velocity on friction coefficient at 60 mN preload. (**b**) Effect of normal load on friction coefficient at 20 mm/s scanning velocity. (**c**) Tactile perception of yarn weight by *S*(*FFT*^2^) at 60 mN normal load. (**d**) Tactile perception of yarn weight by *S*(*FFT*) at 20 mm/s scanning velocity. (**e**) Microscope photographs of six cotton canvas fabrics with different yarn weights. (**f**) Yarn weight tactile discrimination by *kv* and *kw*.

Six cotton canvas fabrics with different yarn weight ranging from 3.5 to 15 oz/yd^2^ slid against a steel ball reciprocatingly with increasing velocity and normal load as shown in Figure 4. Firstly, the friction coefficient curves with increasing sliding velocity from 5 to 40 mm/s and a fixed normal load of 60 mN are shown in Figure 4a. It shows that the COF values increase as the velocity increases, which demonstrates that the fabric yarn weight can be discriminated by conducting the friction coefficient with increasing velocity and fixed normal load. However, the friction coefficient curves with increasing normal load from 20 to 100 mN and a fixed sliding velocity of 20 mm/s presented a lot of overlapping points, which is indistinguishable as shown in Figure 4b. 

In this yarn weight tactile perception study, we modified the roughness tactile discrimination method on fabric materials properties identification by conducting *S*(*FFT*^2^) the power spectra integral analysis with increasing velocity. The rougher surfaces induced a wider range of *S*(*FFT*^2^) values, as it was shown in Figure 4c, 15 oz/yd^2^ fabric indicated a *S*(*FFT*^2^) value ranging from 1.731 × 104 to 8.258 × 104 when the velocity increasing, while the fabric with a yarn weight of 3.5 oz/yd^2^ produced a smaller range of *S*(*FFT*^2^) values from 0.77 × 104 to 1.324 × 104. Furthermore, it is obvious that the slope of the curves is all different, and a larger yarn weight fabric indicates a larger slope value. Thus, the slope value of the *S*(*FFT*^2^) curve with increasing velocities is defined as *kv*, based on the least-squares method. There is a clear monotonic relationship between *kv* and the yarn weight of fabrics, because the fabric surface roughness is directly influenced by the yarn weight values. Then, *S*(*FFT*) frequency spectrum was conducted with an increasing normal load applied on the interface of fabrics in different yarn weight, as it is shown in Figure 4d, six different curves—which represent six different hardness values as we presented in the previous study [14]—are able to discriminate the soft fabrics from the hard ones. 

The slope values of these curves show an obvious correlation with the measured hardness values as shown in Table 1. The harder fabric reveals larger range of *S*(*FFT*) values and softer one has a smaller range, such as 15 oz/yd^2^ are ranging from 1.119 × 103 to 2.631 × 103 during the normal load increase from 20 mN to 100 mN, while the 3.5 oz/yd^2^ fabric produces a smaller range of *S*(*FFT*) which is 0.515 × 103 to 0.806 × 103 as shown in Figure 4d. Thus, *kw* is able to describe the hardness of different yarn weight fabrics with increasing normal load. Figure 4e shows the morphology of the six fabrics with yarn weight from 3.5 to 15 oz/yd^2^ and Figure 4f gives the *kv* and *kw* values of different yarn weight fabrics, which are generated from the above curves. The area of square bars is formed by the error bars of the *kv* and *kw* values, which are obviously separated from each other and provide a linear distribution. This indicates that *kv* and *kw* can efficiently distinguish fabrics with different yarn weights by characterizing the roughness and hardness of these fabrics, which is obviously an accurate and reliable approach in identifying the fabric yarn weight. It also shows great of advantages such as time efficiency and a uniformly experimental process compared with human fingertip tactile perception.

### 3.4. Weave Pattern Tactile Perception

**Figure 5:**
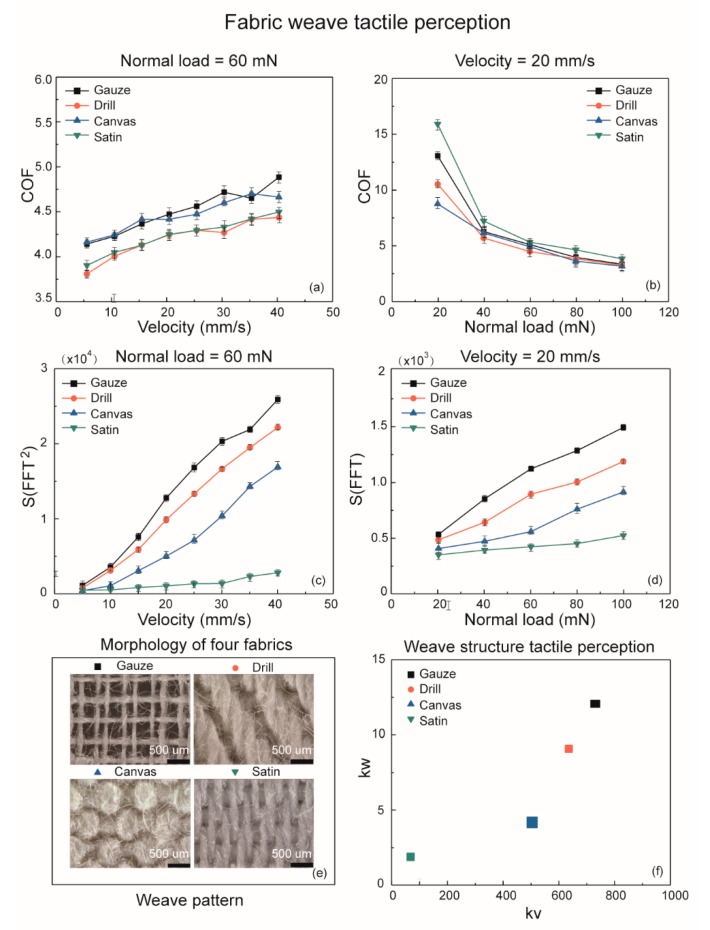
Frictional properties and tactile perception of four fabric weave patterns as a function of velocity and normal load on cotton fabrics. (**a**) Effect of velocity on friction coefficient at 60 mN preload. (**b**) Effect of normal load on friction coefficient at 20 mm/s scanning velocity. (**c**) Roughness tactile perception of four weave patterns by *S*(*FFT*^2^) at 60 mN normal load. (**d**) Hardness tactile perception of weave patterns by *S*(*FFT*) at 20 mm/s scanning velocity. (**e**) Microscope photographs of four cotton canvas fabrics with different weaves. (**f**) Fabric weave pattern tactile discrimination by *kv* and *kw*.

In this section, four pieces of fabric with different weave patterns—satin, canvas, drill, and gauze—were tested. We chose the same weft yarn sliding direction for all the samples as the warp direction vary from the weft direction in different weave patterns. Then, the frictional and spectrum analysis results were shown in Figure 5.

**Figure 6:**
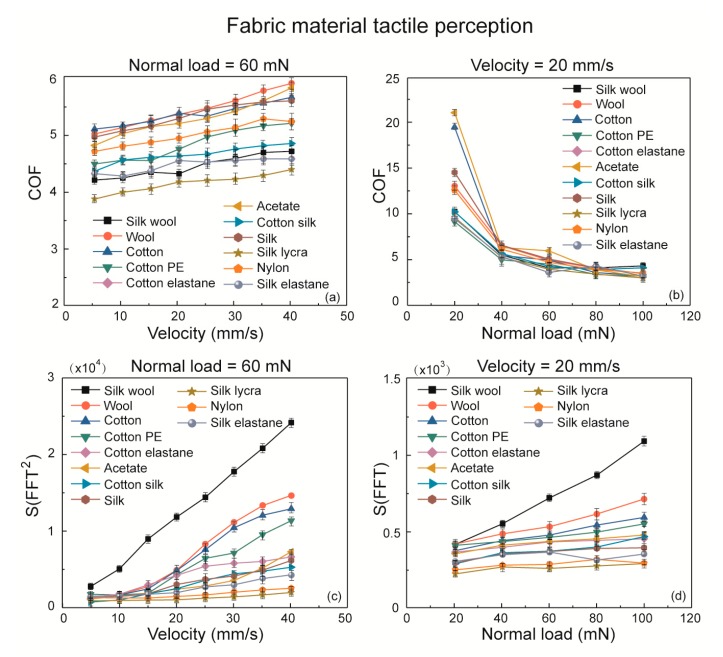
The frictional properties and tactile perception of 11 common fabric materials as a function of velocity and normal load. (**a**) Effect of velocity on friction coefficient at 60 mN preload. (**b**) Effect of normal load on friction coefficient at 20 mm/s scanning velocity. (**c**) Roughness tactile perception of five pure materials and six mixed materials textile fabrics by *S*(*FFT*^2^) at 60 mN normal load. (**d**) Hardness tactile perception of 11 textile materials by *S*(*FFT*) with velocity equals to 20 mm/s.

The COF curves in Figure 5a indicates a significant of overlaps as the velocity increases from 5 mm/s to 40 mm/s and the phenomenon of overlapping also appears when the normal load increased from 40 to 100 mN in Figure 5b. It is such an obvious disadvantage for the frictional analysis on fabric weave patterns, because the frictional properties on fabric weave pattern are not only affected by weave structures, but also other factors such as yarn weight, spatial density, and material hardness. However, when we conducted the spectrum analysis by *kv* and *kw* in frequency domain, it revealed significantly different curves with different roughness and hardness information from the four different weave patterns as shown in Figure 5c,d. Thus, the distribution of weave patterns by *kv* and *kw* is shown in Figure 5f, which presents a clearer distinction and superiority on fabric identification compared with the fictional discrimination by COF. This method of spectrum analysis by *kv* and *kw* values is proven to be an efficient identification method on yarn weight and weave pattern. Thus, we are able to investigate the comprehensive identification of common fabric material properties by conducting the yarn weight and weave pattern identifications in *kv* and *kw* values as it is shown in Figure 6.

### 3.5. Fabric Material Tactile Perception

Eleven textile fabrics made from pure material of cotton, silk, wool, acetate, nylon; and mixed materials of cotton and silk, cotton and polyethylene, cotton and elastane, silk and wool, silk and elastane, and silk and lycra were studied in the research. Frictional properties and spectrum analysis are all present in Figure 6. 

**Figure 7:**
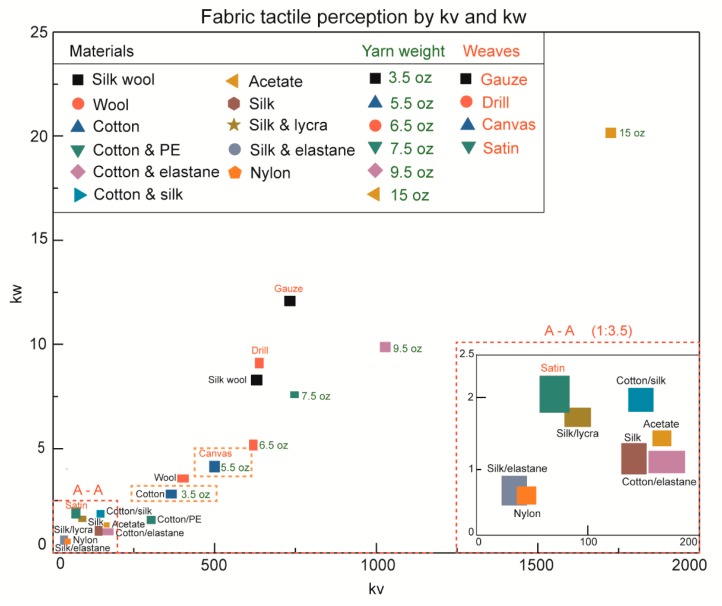
Tactile discrimination of 21 common textile fabrics on the physical properties of material, yarn weight and weave pattern, while they are distributed by our approach of *kv* and *kw* values. An enlarged window (A-A 1:3.5) illustrates the tactile perception overlaps of some textile fabrics.

It can be seen that some fabric substrates with rougher surface and harder material property indicate higher COF values in the Figure 6a,b. However, a majority of the friction coefficient values turn out to be in overlaps, which makes the tactile discrimination of fabric material unable to be proceeded. In the spectrum analysis of *S*(*FFT*^2^) and *S*(*FFT*) curves in Figure 6c,d, even though there are many points reveals to be similar values of *S*(*FFT*^2^), such as wool and cotton substrate at velocity of 20 mm/s normal load of 60 mN, the value of *S*(*FFT*^2^) are 4.441 × 104 and 4.420 × 104, silk and nylon with velocity of 20 mm/s and preload of 40 mN, and so on, the slopes of the curves of *S*(*FFT*^2^) and *S*(*FFT*) are significantly different and distinguishable by comparing with the *kv* and *kw* error bars shown in Figure 7. We present a comprehensive tactile perception system basing on our method of characterization the power spectrum integral by *kv* and *kw* values as the sliding velocity and normal load increase to conduct the fabric material properties identification. The previous results of yarn weight and weave pattern discrimination were combined with fabric material tactile perception by *kv* and *kw*. In total, 21 pieces of fabric substrates were studied as they are present in three columns on the left top of the following figure.

The first two columns are fabric material tactile perception samples with pure and mixed materials, then the third column is yarn weight samples which are labeled in green, weave pattern samples is the last column labeled in red. Moreover, there are 3.5 oz/yd^2^ yarn weight and 5.5 oz/yd^2^ yarn weight cotton canvas fabric samples are marked with blue color dashed boxes, as the same sample used in two tests. From this whole figure comparison, we found that the fabrics with *kw* value ranging from 250 to 2000 appeared to be significantly distinguishable, the square areas which are formed by *kv* and *kw* error bars are clearly separated from each other in the *kv* and *kw* coordinate system. The *kv* and *kw* represents the fabric surface roughness and hardness, respectively. The roughest and softest fabric is the cotton canvas fabric with a yarn weight of 15 oz/yd^2^, which possesses a roughness of 0.951 µm (*Ra*) and a hardness of 85 Shore-o and it gives the largest *kv* and *kw* values of 1732 and 20.1. Then, pure materials of cotton and wool show *kv* and *kw* values larger than most mixed materials such as cotton and silk, cotton and polyethylene, etc.; except silk and wool fabric, which has a *kv* of 618 and a *kw* of 8.3, because this fabric contains ultra-thin yarn of silk of and normal thick yarn of wool as the microscope photo shows in Figure 2, and an unconfirmed plain weave structure of this fabric leads to a small yarn density but rough surface of 0.431 µm in *Ra*, so the *kv* is even higher than pure material of wool. Moreover, the low spatial density of silk and wool fabric surface also generates a larger deformation during the fingertip and steel ball interaction, so it gives a higher measured hardness and a *kw* value as what we conducted in the figure. Then, gauze fabric made from cotton material is supposed to have a small yarn density and reveals large *kv* and *kw* values as it is shown in Figure 7. The fingertip tactile perception results in the weave pattern test are also provided to fit this result. Furthermore, to present a better demonstration of the smooth and hard fabrics with *kw* smaller than 250, such as silk and elastane, nylon, silk and lycra, and cotton and silk. at the bottom left corner section, an enlarged view of A-A (1:3.5) marked with red dashed box is shown on the right side. We found that over 90% of the fabric substrates in the Figure 7 can be discriminated by our new method, expect nylon and silk and elastane fabrics, satin, and silk and lycra fabrics, which revealed a little overlapping at the edges of the error bars.

## 4. Conclusions

In this paper, we explored three aspects of fabric properties—which include yarn weight, weave pattern, and materials—by comparing the human fingertip sensation and the tactile sensor measurement on fabric tactile perception of rough/smooth, hard/soft, and pleasant/unpleasant. Our main objective was to conduct a humanoid identification on fabric material properties with a fingertip-inspired tactile sensor. By mimicking the human fingertip sensation process, a novel method was proposed to characterize the power spectrum integral on friction induced vibration signal and conduct the *kv* and *kw* values of the *S*(*FFT*^2^) and *S*(*FFT*) with increasing sliding velocity and normal load, where the *kv* and *kw* are monotonically related to the roughness and hardness of the fabric surface. Twenty-one different fabrics with various surface textures were significantly discriminated by *kv* and *kw* values, which is proved to be more accurate and objective as compared to the human fingertip sensation or friction coefficient (COF) discrimination. Furthermore, the humanoid identification method of *kv* and *kw* coordinate system successfully solved the instability of experimental on fabric tactile perception which includes the influence of sliding velocity and normal load during interaction with the fabric surface. Such studies provide a new insight for fabric textures and possible object identification applications would be found in textile designs and robotic tactile sensor developments.

## Figures and Tables

**Table 1:** Properties of fabric substrates as function of yarn weight and weave structure.

Name	Yarn Weight of Cotton Canvas (oz/yd^2^)						Weaves			
	3.5	5.5	6.5	7.5	9.5	15	Gauze	Drill	Canvas	Satin
*Ra* (µm)	0.225	0.348	0.382	0.461	0.622	0.951	0.436	0.401	0.348	0.132
Hardness (Shore o)	92.0	90.5	89.0	8.0	87.5	85.0	93.0	87.0	87.5	88.0
*kv*	366	503	620	718	1028	1732	728	633	503	64
*kw*	2.67	4.15	5.13	7.86	9.81	20.1	12.1	9.11	4.15	2.08

**Table 2:** Properties of fabric substrates as function of material.

Name	Pure Materials					Mixed Materials					
	Silk	Acetate	Cotton	Wool	Nylon	Cotton and Polyethylene	Cotton and Elastane	Cotton and Silk	Silk and Lycra	Silk and Elastane	Silk and Wool
*Ra* (µm)	0.179	0.194	0.286	0.310	0.042	0.227	0.201	0.174	0.104	0.038	0.431
Hardness (Shore o)	90.5	91.0	93.5	94.0	88.5	91.5	90.5	92	91.5	89	95
*kv*	140	161	366	385	39.1	299	163	143	88	30.1	618
*kw*	1.16	1.43	2.67	3.58	0.631	1.71	1.10	1.95	1.71	0.708	8.30

## Supplementary Materials

Supplementary Materials can be found at http://www.mdpi.com/1424-8220/18/6/1820/s1, Figure S1: A participant exploring a set of stimulus by fingertip in the light tent condition. The inset shows the fabrics stimulus.
