# Supplementary material for: Humanoid Identification of Fabric Material Properties by Vibration Spectrum Analysis
Source: Sensors (Basel). 2018 Jun 5;18(6):1820. doi: 10.3390/s18061820 (PMC6022104; doi:10.3390/s18061820)
Supplement: Supplementary file 1 [file sensors-18-01820-s001.pdf]

Supplementary Material

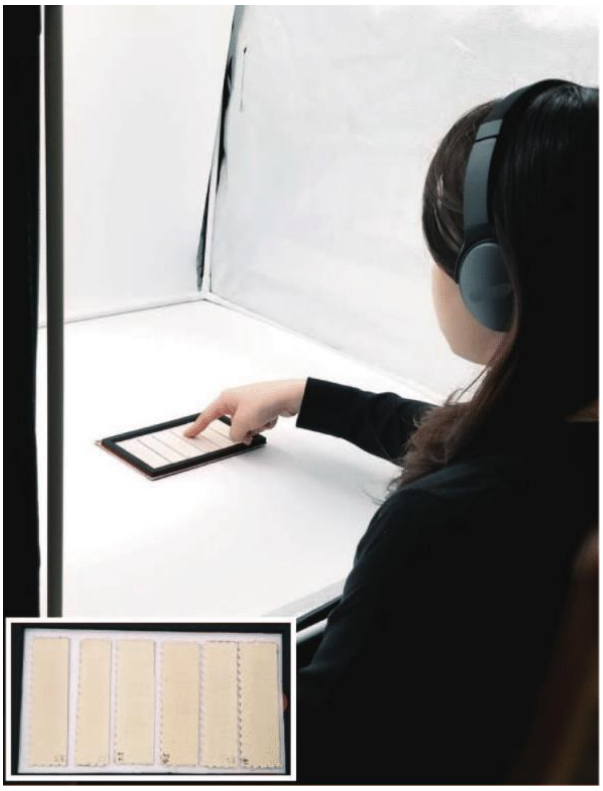

Figure S1. A participant exploring a set of stimulus by fingertip in the light tent condition. The inset shows the fabrics stimulus.
